# Medical Association of Central New York

**Published:** 1905-01

**Authors:** 


### SOCIETY PROCEEDINGS.

### Medical Association of Central New York.

MINUTES of the thirty-seventh annual meeting of the Medical Association of Central New York, held in Star Hall, Masonic Temple, Rochester, N. Y., October 18, 1904, reported by Charles A. Greenleaf, M.D., secretary; Dr. Charles A. Van Der Beek, president.

The president, Dr. Charles A. Van Der Beek, took the chair and called for the report of the secretary. The secretary stated that heretofore this report has been printed in the transactions, and it has been customary in the past to omit it. For that reason he moved that the reading of the secretary's report be omitted. Carried.

The next order of business was the appointment of committees, which the president appointed as follows: on entertainment: C. A. Greenleaf, Rochester; C. A. Huber, Rochester; Wm. M. Brown, Brighton; on reception: E. B. Angell, Rochester; John O. Roe, Rochester; J. W. Magill, Rochester; on credentials: A. L. Benedict, Buffalo; J. Pope Delaney, Geneva; F. H. Stephenson, Syracuse; on business: William B. Jones, Rochester; Sargent F. Snow, Syracuse; Charles E. Congdon, Buffalo; on ethics: A. H. Brown, Auburn; Floyd S. Crego, Buffalo; William A. Howe, Phelps; on publication: William C. Krauss, Buffalo; the secretary; the treasurer.

The report of the treasurer, Dr. C. M. Brown, was then presented as follows:

| | | |
|---|---|---|
| 1903. | DR. | |
| Sept. 22 | —To cash on hand | $242.97 |
| | Dues collected during the year | 226.00 |
| | | $468.97 |
| | CR. | |
| 1903. | DISBURSEMENTS. | |
| Sept. 22 | —W. C. Krauss | $ 3.00 |
| Sept. 22 | —C. A. Greenleaf, salary | 10.00 |
| Oct. 10 | —F. W. Parkhurst, stenographer | 32.23 |
| Oct. 10 | —Gillies Litho. Co | 9.50 |
| Oct. 15 | —Gillies Litho. Co | 25.00 |
| Dec. 24 | —C. A. Greenleaf | 3.80 |
| 1904. | | |
| April 4 | —C. A. Greenleaf | 15.00 |
| Aug. 25 | —A. T. Brown Co | 79.90 |
| Sept. 12 | —Gillies Litho. Co | 4.85 |
| Oct. 17 | —Envelopes, postage, exchange, etc | 14.37 |
| | | $197.65 |
| Oct. 18, 1904 | —Balance on hand | $271 32 |

The report was received and ordered to be placed on file.

The report of the committee on credentials was deferred until afternoon.

The committee on the revision of the constitution and by-laws was not ready to report, and that report also was deferred to a later hour in the meeting.

The president's address was then given by Dr. Charles A. Van Der Beek. (See p. 275, December issue of the JOURNAL.)

Dr. FRANK H. STEPHENSON read a paper entitled, Toxemia and infections as causes of insanity. (See p. 362.)

### AFTERNOON SESSION.

The president: The first order of business is the election of officers, and I appoint Dr. Loveland, of Syracuse; Dr. Howe, of Ontario, and Dr. McDowell, of Rochester, as tellers. A full list of officers is to be elected this year: a president, two vice-presidents, secretary, and a treasurer, and the first in order is the president.

Dr. HENRY L. ELSNER, Syracuse: The meeting today is one which must emphasise the fact that the Central Medical Association has become an important factor in medical education in the state of New York. Comprised, as its membership is, of those who belong to many county societies, made up as it, therefore, must be of men who stand for much in the profession, wielding, as this association does, an enormous influence for good throughout the central, southern and western parts of the state, it is incumbent upon us to elect as its president a man who typifies everything which is ennobling in the medical profession; a man who, when he occupies the chair, will stand for something; a man who has done good work within the ranks, one whose name is known beyond the limits of the counties which go to make up the membership of this association. The present vice-president of this association is such a man, a man who has done thorough and original work in the profession, one whose name is known beyond the borders of New York state; indeed, is recognised as authority on internal medicine. It gives me the greatest pleasure, therefore, to put in nomination for president, Dr. Charles G. Stockton, of Buffalo, and I sincerely trust he will receive the unanimous support of the association.

Dr. F. S. CREGO, Buffalo: I desire to second the nomination of Dr. Stockton. I had intended to nominate him, but Dr. Elsner forestalled me. However, I concur in all that Dr. Elsner has said. I am Dr. Stockton's near neighbor and see a great deal of him in hospital and other professional work. He is in every way worthy of the enconiums pronounced, and I trust he will receive a unanimous vote.

Dr. Edward B. Angell, Rochester: I move that the secretary be instructed to cast one ballot for Dr. Stockton for president for the coming year. Carried; whereupon the secretary complied with the order, and the president declared Dr. Stockton duly elected.

Dr. J. C. Shoudy, Syracuse, in an appropriate speech nominated Dr. D. M. Totman, of Syracuse, for first vice-president.

Dr. Nathan Jacobson, Syracuse, presented the name of Dr. F. H. Stephenson, of Syracuse, for the position of first vice-president.

Dr. F. H. Flaherty, Syracuse, seconded the nomination of Dr. Totman.

Dr. Edward B. Angell seconded the nomination of Dr. Stephenson.

Dr. Crego also seconded the nomination of Dr. Stephenson.

A ballot was then taken and the vote resulted as follows: Stephenson, 21, Totman, 51; whereupon the president declared Dr. Totman duly elected first vice-president.

The president then called for nominations for second vice-president and Dr. N. H. Soble, Elmira, nominated for second vice-president, Dr. Lucius A. Smith, of Palmyra. Dr. Young seconded the nomination, and, on motion, the secretary cast the ballot of the association for Dr. Smith, whereupon the president declared him duly elected.

The president next called for nominations for secretary for the term of three years. Dr. Howe placed Dr. C. A. Greenleaf in nomination, which was duly seconded.

The president was directed by vote to cast the ballot of the society for Dr. Greenleaf for secretary for the ensuing three years, and he declared Dr. Greenleaf duly elected.

The president called for nominations for treasurer.

Dr. T. O. Tait, Rochester, nominated Dr. C. M. Brown, which being seconded, the president declared the nominations closed, the secretary cast one ballot for Dr. Brown for treasurer, and the president declared him duly elected for three years.

The secretary then read the report of the committee on revision of the constitution, giving the following proposed changes:

Article 2, to read: The association shall meet annually on the third Tuesday of October, the meetings to be held in Rochester,

Buffalo, and Syracuse in rotation, and in Auburn by special invitation.

Article 3: To have Allegany, Cattaraugus, Chautauqua, Chemung, and Niagara added.

Article 8, to read: It shall be the duty of the secretary to keep an accurate record of the proceedings of the meeting of the association, to report all communications, to keep a list of the names of all members and delegates, and to send notices to members at least ten days previous to each meeting.

Article 10, to read: To "take charge of" in place of "procure."

Article 12, to read: "That may be required," in place of "it may see fit."

Article 15: To be stricken out as superfluous.

Article 16, to read: This association shall have power by vote at its regular meetings to expel any member for unprofessional conduct, or for acts derogatory to the honor and good name of the profession.

Article 19, to read: Any member of any county society may have papers read for them by delegates on whatever subject they may choose.

On motion of the treasurer a clause was added, providing for dropping members who may allow their dues to lapse for four years, after a proper hearing.

On motion of Dr. A. A. Young, Newark, the words "to Auburn by special invitation" were changed, so as to read, "any other place by special invitation."

After debate a vote was taken and the report adopted, the president announcing that the committee is continued for another year.

New members were elected as follows: F. H. Parker, Auburn; T. E. O'Brien, Auburn; W. C. McKeeby, Syracuse; I. H. Rosenthal, Rochester.

Dr. J. Henry Dowd, Buffalo, read a paper entitled, Inflammation—its control.

On motion of Dr. W. J. Herriman, Rochester, the president appointed a committee to give appropriate expression of sympathy to Dr. Louis A. Weigel, of Rochester, on account of his recent surgical misfortune. The committee, consisting of Drs. Herriman, Rose, and Totman, subsequently reported as follows:

"The officers and members of the Medical Association of Central New York, assembled in their thirty-seventh annual meeting, desire to convey to their associate member and former president, Dr. Louis A. Weigel, of Rochester, their sincere sympathy in his recent misfortune. The loss he has sustained was received in his unselfish work in the cause of medical science, and this fact accentuates the sorrow with which his professional co-workers learned of his affliction. Let this message convey to him our very fond wishes and best hopes for the future."

(Signed) W. J. HERRIMAN,
L. W. ROSE,
D. M. TOTMAN.

On motion of Dr. YOUNG, the report was adopted by a rising vote. The memorial was ordered engrossed and sent to Dr. Weigel.

Dr. FREDERICK H. SAWERS read a paper entitled, Quarantine.

Dr. ALBERT C. SNELL, by invitation, read a paper entitled, The magnet in eye work—its use in two cases.

Dr. A. L. BENEDICT submitted without reading his paper entitled, A statistical study of one hundred and fifty cases of movable kidney.

The secretary from the committee on entertainment reported that the annual banquet will be held in the grill room of the Masonic Temple, at 8.30 p. m. The price of each plate for this dinner will be one dollar.

The president: The next meeting will be held the third Tuesday in October, 1905, in the city of Buffalo. I wish to thank the members of the association and our guests for the courtesy and kindness which they have shown in assisting me in conducting the affairs of this meeting. The association is now adjourned to meet next year in Buffalo.

